# Lithuanian health care in transitional state: ethical problems

**DOI:** 10.1186/1471-2458-5-117

**Published:** 2005-11-09

**Authors:** Irayda Jakušovaitė, Žilvinas Darulis, Romualdas Žekas

**Affiliations:** 1 Department of Philosophy and Social Sciences, Kaunas University of Medicine, Mickeviciaus 9, 44307 Kaunas, LT, Lithuania; 2 National Board of Health of Lithuania, Gedimino 53, Vilnius, LT, Lithuania

## Abstract

**Background:**

Throughout the economic and political reforms in post-communist countries, significant changes have also occurred in public morality. One of the tasks of the Lithuanian health policy is to create mechanisms for strengthening the significance of ethical considerations in the decision-making processes concerning health care of individuals and groups of individuals, as well as considering the positions of physicians and the health care system itself in a general way. Thus, health care ethics could be analyzed at two levels: the micro level (the ethics of doctor-patient relationships) and the macro level (the ethics of health policy-making, which can be realized by applying the principles of equal access, reasonable quality, affordable care and shared responsibilities). To date, the first level remains dominant, but the need arises for our attention to refocus now from the micro level to the patterns of managing and delivering care, managing the health care resources, and conducting business practices.

**Discussion:**

In attempting to increase the efficiency of health services in Lithuania, a common strategy has been in place for the last fifteen years. Decentralization and privatization have been implemented as part of its policy to achieve greater efficiency. Although decentralization in theory is supposed to improve efficiency, in practice the reform of decentralization has still to be completely implemented in Lithuania. Debates on health policy in Lithuania also include the issue of private versus public health care. Although the approach of private health care is changing in a positive way, it is obvious that reduced access to health services is the most vulnerable aspect. In the Lithuanian Health Program adopted in July 1998, the target of equity was stressed, stating that by 2010, differences in health and health care between various socio-economic groups should be reduced by 25%.

**Summary:**

The restructuring of health care system in Lithuania should be based on a balance between decentralization and centralization, and between public and private health care sectors. Successful transition requires a balanced role of the government. Today it is obvious in Lithuania that continuous encouragement to make sacrifices was not enough to induce the system to function well, and in an ethical manner.

## Background

In undertaking any health care reform, it is important to clarify the ethical problems they may engender [1-4]. Lithuania as well as Eastern and Central European countries in transition have postulated the importance of ethical considerations in changes in the health sector. In Lithuania, ethics has traditionally focused on the problems of the right and wrong conduct of individuals [5,6]. Transition to a more technological world confronts us with a new kind of human behavior, namely the management of collective systems. Today institutions, not just individuals, practice health care. Thus, we need to reorient health care ethics towards the issues of organizational activity and management instead of emphasizing the traditional discussions and moralizing. Behavioral changes must occur, but this can only be achieved within the framework of institutional and political support to ensure equal access, reasonable quality, and affordable care and shared responsibility in health care. It is a paradox that patients and physicians have to defend their rights against the advent of very complex health care systems, the aim of which should be to help them. Thus, we need to speak about the moral problems of health care system in the context of individual (micro) and social (macro) ethics [7]. Only synchronous functioning of these levels can ensure an ethical solution of the problems. When redistributing the gross public product, healthcare in Lithuania receives 3,6% of the gross national product. Despite this – and according to a summary of the achievements of the system – the effectiveness of the health care system of Lithuania occupies 52^nd ^position in the global rating out of 191 countries. However, our country is only in 131^st^–133^rd ^position with regard to the equity of financing of the system [8]. Such dissonance may not remain for long, since deficit in the financing sector will result in a crisis of human resources, lack of infrastructure and progress in the field of medicine, and will retard the reduction of health inequalities in Lithuania.

After the restoration of its independence in 1989, Lithuania inherited a centralized system that mainly delivered inefficient health care management and resource allocation. It opted for restructuring and decentralization as strategies that would to increase the efficiency of our health services. Decentralization of the health care system was achieved by segregating primary health care (family physicians), secondary health care (physicians – specialists), and tertiary health care levels (high specialization university clinics). The development and reformation of primary health care was seen as a key factor in the entire health care reform [9]. However, the experience of other countries shows that in practice, decentralization does not necessarily enhance efficiency. According to Reich [10], first "a local community still has a range of preferences within it, so that problems of allocative efficiency remain. Second, local government can still be secretive and unrepresentative, so that voter demands are not reflected in local bureaucratic decisions. Third, decentralized government can still be highly bureaucratic government, with high degrees of technical inefficiency, especially if local units have limited managerial capacity and expertise. Fourth, the small scale of decentralized units can produce a loss in economies of scale. In short, decentralization in practice creates multiple problems that may overwhelm any efficiency gains expected in theory". All these problems are obvious in Lithuania.

Many expectations are associated with the emergence of the private health care sector in Lithuania. The appearance of the private sector has raised many debates. Although the approach of private health care is changing in a positive way, it is obvious that in the private health care decreased access to health services is the most vulnerable, as the experiences of other countries have shown [11-14]. We agree with Segall's statement [15] that "privatization in health care does not lend itself to a quick technical fix. It is a complex process, essentially political and ethical in character, and involves the interplay of a number of considerations among which are not only those of equity and efficiency".

The aim of this article is to define the main social ethical problems in Lithuanian health care system in transition and to discuss the possible solutions to these problems in the context of the experiences of other countries. This has involved analysis of original research papers and official documents of Lithuanian health care system combined with the authors' personal opinion.

## Discussion

All countries of Central and Eastern Europe have now expressed their wish for a total change in their health care systems. Changes in these countries include: the introduction of market economy mechanisms in health care, an increased focus on population needs in guiding health care systems, and the possibility of introducing a more general type of care at the primary level [16]. Ethical issues should be taken into account during the process of the implementation and evaluation of these changes in the healthcare system.

### Balance between decentralized and centralized management in health care

An attempt to increase efficiency of our health services has been a common goal over the last fifteen years in Lithuania. Decentralization was one of the strategies to achieve it. The Lithuanian National Board of Health encourages the decentralization and privatization of the institutions of primary health care and the centralization of the institutions of secondary health care.

From 1994 to 1995, one of the political decisions was to devolve health care services by shifting administration from the Ministry of Health to the ten counties. At the same time, funding responsibilities were moved from the Lithuanian Ministry of Health to the State Sickness Fund. The counties are in charge of the enforcement of the state health programs in their respective regions. The municipalities are responsible for providing primary health care to their local population and are engaged in running small and medium size hospitals within their localities. Moreover, municipalities have a wide range of responsibilities in the implementation of local health programs and the improvement of public health activities. Decentralization of management transfers responsibility to where the work is actually done, allowing for the search for optimal solutions for the achievement of the results of health promotion in real conditions. In essence, this would mean the development of a new managerial model that would clearly define the rights, responsibilities and accountability of the participants in the health promotion process. Despite increasing cooperation between administrators, providers and consumers, the last remains the weakest in the bargaining process. Municipalities and county administrations do not have enough capacity to plan the services under their responsibility and appear to lack the authority to enforce their decisions. Municipalities responsible for maintaining and developing their respective health care infrastructure do not allocate sufficient resources for this purpose. A serious obstacle to health care reform in Lithuania is a continuing lack of managerial skills and low interest in the application of professional expertise in decision-making, [17]. In addition, municipalities that make decisions related to the development of primary healthcare services impede private capital investment. Municipal Councils that make such decisions demonstrate significant lobbying of state institutions. Lithuanian Law of Health Care Institutions [18] allows permitting or depriving the establishment of private health care institutions. Thus, there is an obvious gap between the aims of the healthcare reform policy and the expectations of the patients.

The situation of centralization in health care is much the same. Although the centralization of the institutions of secondary health care in Lithuania was successful in improving the quality of services, many issues remain unsolved. The annual report of the National Health Council states that the network of stationary healthcare institutions is excessive and irrational. Due to the lack of medical technologies and human resources, healthcare institutions of a lower level cannot ensure quality of healthcare. At present, certain hospitals perform one or two complex operations or procedures per year. In the absence of a sufficient number of operations or procedures, discussions about the quality of service become complex. The report indicates that Lithuania should adopt the model that proved to be optimal in the world and Europe, i.e. merging of hospitals rather than closing them. This would preserve human resources, modern technologies, and the experience of the merged hospitals. The main issue of the opponents of this model is with the assurance of the accessibility of healthcare services in a geographical/territorial sense [19].

It is very difficult to explain to people in the regions that although they have mandatory health insurance, they cannot sometimes get one or other of the services they had before. This situation raises social problems as well; not every patient can afford paying for the trip to the needed specialist who works in a higher-level institution in the district center or some big city. But the problem is more complex. There are over 29,990 hospital beds in Lithuania. Comparing hospital cases per 1000 inhabitants with the same in Sweden, it is becoming clear that in Sweden, 3600 hospital beds would be enough for the same treatment [20]. This situation exists because a lot of patients are treated in Lithuanian hospitals, while in Sweden and other countries many more would be treated in out patient departments (LOR, eye, nervous, many mental and other illnesses). In addition, our secondary health service is highly institutionalized in Lithuania; there is no out-patient care or nursing [21]. This model of treatment raises the threat of ageism (disturbance of human dignity, the "slippery slope" tendency).

### From management to ethics

Improvement of management is one of the preconditions for tackling ethical problems in health care. The improvement of administration/management would ensure better satisfaction of the needs of patients for the same expenditure. However, the actual administration of health care institutions does not meet the patients' needs. According to the data of different surveys, both physicians and patients unanimously agree that the existing administration of health care institutions is inappropriate – >20% of respondents claimed they were unhappy with the quality of treatment, and ~30% of respondents were not satisfied with the attention paid and the quality of treatment in public health care institutions, while <5% of the respondents indicated difficulty in registration, bad quality of services and not insufficient attention paid in private health care. About 60% of patients were not satisfied with primary health care administration, mainly stressing the appointment system as not appropriate, rudeness of the employees at the reception desk, insufficient time for patient's examination, and a lack of effective distribution of functions. Physicians, on their part, admit that usually health care institutions have no "team" with clearly distributed responsibilities. Thus, a physician at the same time fills the papers and works as a psychologist, social worker, and service provider. The majority of physicians and patients have pointed out the shortage of social workers and their insufficient qualification [22]. The results of the qualitative study helped to identify the main reasons for patients' dissatisfaction, which were have divided into three levels:

1. the system level (dissatisfaction with the health care reform, bureaucracy, difficulty in getting the specialist, and high cost of services),

2. the institutional level (deficiencies in provision and quality of service, long queues, waiting, lack of medical equipment, and inadequate quality of the health care service),

3. the individual level (deficiencies in physicians' attitudes and skills and work, lack of attention, information, and responsibility, and negligence and rudeness).

These three groups indicate the level of responsibility for issues to be addressed. So, we need to speak not only about the problems of individual ethics, but also about moral problems of health care systems and organizational ethics [23]. Changes in the management of primary health care increase patient satisfaction with the doctor. The results of the population survey performed in Lithuania revealed that 69,9% of the respondents were satisfied with their primary care doctor [24]. A similar situation exists in Estonia, where 68% of patients were quite satisfied or very satisfied with their general practitioner or primary health care doctor [25]. Qualitative decision-making in primary health care should help to avoid many ethical conflicts.

### Reimbursement of medicines – the right to quality medications

Another problem pointed out by both physicians and patients is the quota of reimbursed medicines and services. Until 1990, the entire pharmaceutical sector was state-owned. Medicines were subsidized by the state. In 1991, Lithuania decided to harmonize its standards with those of Western Europe, which favored the opening of the Lithuanian market to more expensive drugs produced in the European Union. At the same time, it has prohibited cheaper imports from the former USSR and other countries, as these did not meet European GMP standards. Because of the strong lobbyism of rich manufacturers of brand-name medicines, it is difficult for generic medicines to take a stronger position within the market.

According to the data of the State Sickness Fund, the expenditures of Lithuanian population for medicines in 2003 were ~65%, while the expenditure of the State Sickness Fund for reimbursed medicines was 35% [19]. Drugs are delivered free to the in-patient sector, but the reimbursement system for the drugs prescribed in the out-patient sector is complicated. Pharmaceutical companies, when operating through chief specialists in certain fields (e.g. chief pulmonologists, chief cardiologists, etc.), as well as through ordinary physicians, influence the patients' attitude, orientating towards treatment with the newest and the most expensive medicines. The state, on its part, having limited resources, attempts to regulate this process (through the procedure of reimbursement for the medicines, through the introduction of drug prescription quotas for physicians and healthcare institutions, etc.), so that the financial possibilities are not exceeded. Proposals were made for a more frequent use of generic medicines, which are cheaper. This brings about significant ethical conflicts between the Ministry of Health and the pharmaceutical companies, as well as between physicians and patients. Physicians are now in a paradoxical situation where having more patients and prescribing more subsidized medicines means the institution might sink into debt. Physicians working in primary health care institutions arrive at a paradoxical conclusion: the best physician is the one whose patients are healthy and rarely visit the institution; therefore they do not require subsidized medicines and expensive examinations. It is not in the interests of the health care institution to have the patients visit the institution, since the less work they do, the more money is allocated to the institution [22].

With its relatively small market of medicines and underdeveloped system of reimbursement, Lithuania can hardly expect the lowest prices of all reimbursed medicines compared with other countries of the EU. An increase in the population's extra pay for reimbursed medicines results in a decrease in the accessibility of new and effective medications [19]. This is an important problem of social ethics.

There has been a lack of surveys in Lithuania needed to estimate for common drugs usage. Every country has its own specificity. The comparative study performed among Estonian and Finnish general practitioners to evaluate the need for common drugs showed that different therapeutic traditions influence the list of essential medications [26].

### Private versus public health care

The period of 1993–1994 was marked with public debate on the issues of private versus public health care institutions in Lithuania. The founders of the institutions that belong to the Lithuanian National Health System (LNHS) are the State, the Ministry of Health, counties, municipalities, and private persons (independent contractors). All these institutions receive financing from the Sickness Funds. There are healthcare institutions whose founders are private persons who have not signed contracts with the Sickness Funds, and thus the services of such institutions are fully paid by the patients. In Lithuania, there are few private healthcare institutions that do not belong to the LNHS. The number of rich people in Lithuania is also low, and people, having paid for obligatory health insurance are unwilling to pay for the services a second time. Private healthcare institutions that belong to the LNHS function much more effectively.

The approach to private health care is changing. According to a survey conducted in October 1995, <25% of health professionals and politicians stressed the private health provision and the market in general as being of future importance in planning and resource allocation in the Lithuanian health sector [19]. Since 1996, the possibility of choosing the general practitioner voluntarily for the society members was provided. When the law of health insurance was submitted, their conditions to get the financial support from the Sickness Founds and to establish the private institutions of family doctors have appeared, although the beginning was rather slow. The European Union PHARE project that took place in 1999–2000 has given a more prompt impulse to the reform. In 1999 and 2000, the National Health Board of Lithuania accepted the resolutions, which claimed the agreement to decentralize and privatize the institutions of primary health care [27]. Unfortunately, the rate of decentralization and the establishment of private general practitioners (GP) have decreased. The heads of public health care institutions had not felt the competition of private GPs at first. Only when the mostly active and perspective doctors have moved to the private institutions taking patients "with them" did the budgets of institutions decline significantly, and there were statements that the whole health care system was being damaged. The appearance of the elements of a market in health care delivery was considered to be chaotic. Municipalities have the right to grant or to refuse the role of the founder of service provider. All service providers are being distributed into "homeys" and "strangers". The boards of municipalities are tending to set the priorities for public institutions and will never let private institutions be established in the more settled areas, where the activity of the institution would be rather profitable (cost-effective). The main reason, they claim, is that there is a state-owned institution in this area already. In this way the municipality plays the main role of market regulation. This was found to have very negative consequences for the coming of private capital into the health sector. It also reduces private initiatives and enterprise, limits citizens' choices of health care providers, although this is based on – and ensured by – certain laws. According to representative survey data, the reason given is that 87,7% of Lithuanians use public health care services, only 12,3% used state health care services, and 12,3% visited private health care clinics or hospitals that had no agreements with the Sickness Funds, requiring full coverage for their services from the patients [24]. However, in some municipalities that have signed agreements with the Sickness Funds, 40% and even more of the population chose private providers of healthcare services. According to the data of the State Sickness Fund, the number of primary healthcare institutions increased from 306 in 2002 to 322 in 2003. The number of state-owned institutions remained unchanged, while the number of private institutions during this period increased from 108 to 124 [19]. Most people who choose the private health care sector have considerably greater incomes comparing to those who choose public health care sector. Indeed, there is nothing unethical about allowing or even encouraging people who can afford to buy their own health care, especially if they continue to pay into the public fund. It is unethical to restrict the person's free choice to private health care. As the experience of other countries shows, "health is in an area of ubiquitous market failure. In the rush to harness efficiency gains from the market forces, policy-makers in developing and transition economics should not blindly apply marketization to the health sector. Successful transition requires a balanced governmental role" [14].

### The state strategy of Lithuania for reducing health care inequalities

In many European countries, the principle of equity has governed many health services and policy decisions. Equity is an ethical concept, which means that health care resources are allocated according to needs, health services are received according to the needs, and payment for health services is made according to the ability to pay. It implies access, quality, and acceptability in health services for all [28,29]. Equity in health care cannot mean total equality of health, but it can mean the reduction and, ultimately, elimination of avoidable inequalities in health. The experience of developed countries shows that the highest level of health is seen not in the richest countries (societies), but rather in those where the difference of family income between the rich and the poor is the smallest.

The first report on health care inequalities in Lithuania was published in 1998. Data from the mortality register indicates that education, socioeconomic group, and marital status were significant predicting factors in health inequality. Higher level of education, higher income, and urban places of residence gave strong positively correlation with self-reported health status and better health, especially with regard to smoking and alcohol consumption. Large inequalities in neonatal health according to the mother's level of education and marital status were discovered. Finally, socio-economic inequalities were found as having an influence on health care accessibility, with lower socioeconomic status predicating decreased access to the services. Large differences were observed in relation to socio-economic status in other Baltic states. In Lithuania, we face the same problem as in Estonia when the reform of the primary health care was recently evaluated in terms of efficiency, but not in terms of equity [30]. Thus, one of the objectives of the Lithuanian Health Program adopted in July 1998 was to ensure equity in health and health care. The Lithuanian Health Program contains the particular target of equity, which states that by the year 2010, the differences in health and health care between various socioeconomic population groups should be reduced by 25%. Parliament adopted a resolution stipulating that the actions should focus on ensuring equal rights of access to health for all by decreasing health inequalities among rural and urban populations, populations with different education and income levels, and between age groups by active cooperation of the State, local self-government institutions and non-governmental organizations. The following table presents the indicators of socioeconomic inequalities in health and health frequency of monitoring sources [31].

### The problem of illegal (informal) payments

When talking about the topical problems of health care ethics and patients' rights, one cannot avoid the problem of illegal payments, or so-called "under-the-counter" payments. The concept of these payments becomes more problematic. The participants in discussions used such terms as "additional payments", "gratuities", and "gifts". According to the Lithuanian Civil Code, payment that does not exceed the minimum standard of living is not considered to be a bribe. It appears that people perceive a bribe to be payment to a physician in cash, while cakes, champagne, sweets or coffee (the most popular forms of gratuity) are not considered bribes. The non-civic practices thriving in health care ("knowing the right people" and bribery) should be replaced by behavior models based on normative acts.

According to the quantitative sociological research "Patients' rights in contemporary health care" performed in 2002 by the Bioethics Society and the Center of Civil Initiative, many physicians think that the reasons that make people give bribes to physicians are of a more psychological nature [21]. Since neither the physicians nor the patients indicated a case where the physician tells the patient the amount of money that has to be paid prior to surgery or some other service, or where patient does not receive the necessary assistance because he or she did not pay the physician personally, it is considered that people usually offer bribes because this makes them to feel more secure. However, the results of the survey performed in Lithuania in 2005 showed that informal payment can lead to better access to higher quality health care [32].

It is obvious that patients do not receive sufficient information about the changes in or the provision of the health care system. Patients do not know under what conditions health care services are free, and for which ones they have to pay. They fear that they will have to pay additionally for certain services, and are often in a quandary over whether they should offer a bribe. There is also a lack of transparency at the highest levels, where the money of mandatory health insurance is distributed. One can hardly find a person who would know how the money from the taxes (including health care fees) is distributed; in other words, people do not know what they can get for the money they have paid in taxes. Corruption manifests itself in the sphere of public purchase and privatization. Cases of gratuities to committee members who can influence contest results are also frequent. It is a public secret that sometimes pharmacy companies give bribes when attempting to have a specific medicine registered or included in the list of subsidized medicines, or when trying to attract the physicians to prescribe a specific medicine.

Is it possible to defeat corruption in the health care system? Corruption is a well-known by-product of governments in rich, poor and so-called developed countries [10]. In Lithuania, the State Sickness Fund follows the National anti-corruption rules and uses anti-corruption measures in health care. We believe that in order to defeat corruption, it is not enough to strengthen the control mechanisms. It is even more important to reduce the incentives to give or take the bribes. While it is impossible to eliminate corruption altogether, it is necessary to take all measures to limit its extent. The experience of other countries shows that formalization of some unofficial fees with careful monitoring of their impact may help to fight corruption. The main strategies within this area should be the full recognition (development of the system?) of patients' rights, simple procedures for complaints, transparent contracts of employment, and targeting those facilities in which collusion among professionals leads to "networked" health care fees [33].

It is important to emphasize that corruption is first of all an economic problem. As the countries of the former Soviet Union and Eastern Europe have made the transition from state control of all aspects of economic life, the evolution of health care markets has been at best erratic, and the need for a better managed transition has become apparent. The existence of informal payments is *prima facie *evidence that publicly set prices are insufficient to induce supply, and that threats of sanctions against providers who do not offer services at these prices are insufficient. The appropriate response for governments of course is to set producer prices, but in times of budgetary squeeze and excess capacity, this is infeasible. Without rationalization of the wholesale side of supply, informal payments will continue to play a major role in resource allocation, and negative effects on equity and access will continue [34]. In Lithuania, only the first steps are being made towards the management of economic measures.

## Summary

Ethical considerations should be taken into account while tackling the problems of health care reform. Over the last 30 years, Lithuanian health care institutions have focused primarily on individuals, paying insufficient attention to the problems of social ethics. The latter refocuses our attention from the micro level to the patterns of management and provision of health care. Balance in the restructuring of the health care system between decentralization and centralization in Lithuania, between public and private sectors in health care, the improvement of management, and the evaluation of these changes help to solve many problems of social ethics not only in the aspect of efficiency, but in terms of equity.

## Competing interests

The author(s) declare that they have no competing interests.

**Table 1:** Indicators of socioeconomic inequalities in health and health care, frequency of monitoring and data sources

**Health and health care indicators**	**Socioeconomic indicators**	**Monitoring frequency**	**Data sources**
**1. Mortality (by gender and age groups): **all causes, cardiovascular diseases, malignant neoplasms, external causes, respiratory diseases, other causes	Place of residence: urban – rural	Annually	Mortality register
	Administrative regions	Every 3 yrs	
	Education	Every 10 yrs, based on census data	
	Marital status		
**2. Life expectancy**	Place of residence: urban – rural	Annually	
	Administrative regions	Every 3 yrs	
**3. Avoidable mortality**	Place of residence: urban – rural	Every 5 yrs	
	Administrative regions		
**4. Low birth weight newborns and stillbirths**	Mothers' education	Every 3 yrs	Newborn register
	Mothers marital status		
**5. Health behavior of adult population (by gender and age groups): **Self-assessed health; outpatient visits per year; stress during the last month; depression; oral health; smoking; alcohol use; nutrition; physical activity; traffic safety	Education	Every 4 yrs	Health behavior Monitoring among the adult Population
	Place of residence: urban/rural	Every 2 yrs	
	Income	Every 2 yrs	
**6. Health behavior of schoolchildren (by gender): **Self-assessed health; outpatient visits per year; stress during the last month; depression; oral health; smoking; alcohol use; drug use; nutrition; physical activity; traffic safety	Family well-being index	Every 2 – 4 yrs	Health behavior monitoring in schoolchildren
	Parents' profession		
	Place of residency		
**7. Health care accessibility**	Profession	Every 3 yrs	Surveys on representative samples of the population
	Education		

## Pre-publication history

The pre-publication history for this paper can be accessed here:


